# Supplementary material for: EnzML: multi-label prediction of enzyme classes using InterPro signatures
Source: BMC Bioinformatics. 2012 Apr 25;13:61. doi: 10.1186/1471-2105-13-61 (PMC3483700; doi:10.1186/1471-2105-13-61)
Supplement: Addtional file 5 — The Java code to format the data files, evaluate and predict. The file enzml_java_code.tar.gz contains the Java code used to format database data to ARFF and XML formats, to execute cross and train-test (jackknife) evaluations and to record evaluation results to database. More information is included in the readme.txt file and the Javadoc files. The code can be used with a MySQL database. To use a different database software, other JDBC drivers might be required. [file 1471-2105-13-61-S5.gz › java_code/enzml2011/doc/uk/ac/ed/inf/enzml/mulan/learn/MulanCrossEvaluator.html]

MulanCrossEvaluator


---


|  |  |  |
| --- | --- | --- |
| **Overview** \| **Package** \| **Class** \| **Use** \| **Tree** \| **Deprecated** \| **Index** \| **Help** | |  |
| **PREV CLASS**   **NEXT CLASS** | **FRAMES**    **NO FRAMES**     **All Classes** |  |
| SUMMARY: NESTED \| FIELD \| CONSTR \| METHOD | DETAIL: FIELD \| CONSTR \| METHOD |  |


---


## uk.ac.ed.inf.enzml.mulan.learn Class MulanCrossEvaluator

```
java.lang.Object
  mulan.evaluation.Evaluator
      uk.ac.ed.inf.enzml.mulan.learn.EvaluatorNoAUC
          uk.ac.ed.inf.enzml.mulan.learn.MulanCrossEvaluator
```

**Direct Known Subclasses:**: TrainTestEvaluator

---

``` public class MulanCrossEvaluator extends EvaluatorNoAUC ```

Class created to execute cross-evaluations and extract individual evaluation
results (not possible from the Mulan class Evaluator, because only averaged
results are returned). Calls `ResultsOutput` to save the results to
database.

**Version:**
:   8 Jun 2010

**Author:**
:   Luna De Ferrari luna.deferrari-at-ed.ac.uk

---

**Constructor Summary**

`MulanCrossEvaluator(MulanCrossExperimenter experimenter,
boolean strict)`


| **Method Summary** | |
| --- | --- |
| `java.util.Vector<uk.ac.ed.inf.utils.database.TableRow>` | `evaluate(int numFolds)`             Evaluates (runs a cross validation) |
| `java.lang.String` | `getArffFilePath()` |
| `java.lang.String` | `getEndTime()` |
| `java.util.Vector<uk.ac.ed.inf.utils.database.TableRow>` | `getEvaluationMeasuresRows()` |
| `MulanCrossExperimenter` | `getExperimenter()`             public Evaluation[] getEvaluations() { return m\_evaluations; } |
| `MulanLearner` | `getLearner()` |
| `java.lang.String` | `getStartTime()` |
| `java.lang.String` | `getXmlFilePath()` |
| `java.util.Vector<uk.ac.ed.inf.utils.database.TableRow>` | `internallyCrossValidate(int numFolds, int seed)`             Evaluates a `MultiLabelLearner` via cross-validation on given data set with defined number of folds. |
| `void` | `setEndTime(java.lang.String endTime)` |
| `void` | `setEvaluationMeasures(java.util.Vector<uk.ac.ed.inf.utils.database.TableRow> evaluationResults)` |

**Methods inherited from class uk.ac.ed.inf.enzml.mulan.learn.EvaluatorNoAUC**

`evaluate`

**Methods inherited from class mulan.evaluation.Evaluator**

`crossValidate, crossValidate, evaluate, setSeed, setStrict`

**Methods inherited from class java.lang.Object**

`equals, getClass, hashCode, notify, notifyAll, toString, wait, wait, wait`

**Constructor Detail**

### MulanCrossEvaluator

```
public MulanCrossEvaluator(MulanCrossExperimenter experimenter,
                           boolean strict)
```

**Parameters:**: `experimenter` -


**Method Detail**

### evaluate

```
public java.util.Vector<uk.ac.ed.inf.utils.database.TableRow> evaluate(int numFolds)
```

:   Evaluates (runs a cross validation)

    :   **Parameters:**: `mlDataSet` -: `numFolds` -

---


### getArffFilePath

```
public java.lang.String getArffFilePath()
```

---


### getEndTime

```
public java.lang.String getEndTime()
```

---


### getEvaluationMeasuresRows

```
public java.util.Vector<uk.ac.ed.inf.utils.database.TableRow> getEvaluationMeasuresRows()
```

---


### getExperimenter

```
public MulanCrossExperimenter getExperimenter()
```

:   public Evaluation[] getEvaluations() { return m\_evaluations; }

---


### getLearner

```
public MulanLearner getLearner()
```

---


### getStartTime

```
public java.lang.String getStartTime()
```

---


### getXmlFilePath

```
public java.lang.String getXmlFilePath()
```

---


### internallyCrossValidate

```
public java.util.Vector<uk.ac.ed.inf.utils.database.TableRow> internallyCrossValidate(int numFolds,
                                                                                      int seed)
                                                                               throws java.lang.Exception
```

:   Evaluates a `MultiLabelLearner` via cross-validation on given data
    set with defined number of folds. The specified number of folds has to be
    at least two. If negative value is specified, the used number of folds is
    equal to number of instances in the data set. Also saves the data to file
    and database.

    :   **Parameters:**: `learner` - the learner to be evaluated via cross-validation: `mlDataSet` - the multi-label data set for cross-validation: `numFolds` - the number of folds to be used **Throws:**: `java.lang.IllegalArgumentException` - if either of learner or data set parameters is null: `java.lang.IllegalArgumentException` - if number of folds is invalid: `java.lang.Exception`

---


### setEndTime

```
public void setEndTime(java.lang.String endTime)
```

---


### setEvaluationMeasures

```
public void setEvaluationMeasures(java.util.Vector<uk.ac.ed.inf.utils.database.TableRow> evaluationResults)
```


---


|  |  |  |
| --- | --- | --- |
| **Overview** \| **Package** \| **Class** \| **Use** \| **Tree** \| **Deprecated** \| **Index** \| **Help** | |  |
| **PREV CLASS**   **NEXT CLASS** | **FRAMES**    **NO FRAMES**     **All Classes** |  |
| SUMMARY: NESTED \| FIELD \| CONSTR \| METHOD | DETAIL: FIELD \| CONSTR \| METHOD |  |


---
